# An Efficient UPLC-MS/MS Method Established to Detect Relugolix Concentration in Rat Plasma

**DOI:** 10.3389/fphar.2022.874973

**Published:** 2022-06-16

**Authors:** Liying Xing, Ya-nan Liu, Hongye Yao, Tingting Wang, Fuchen Xie, Shunbin Luo, Pingping Luo, Shengling Tang

**Affiliations:** ^1^ The First People’s Hospital of Jiashan, Jiaxing, China; ^2^ The First Affiliated Hospital of Wenzhou Medical University, Wenzhou, China; ^3^ The People’s Hospital of Lishui, Lishui, China

**Keywords:** GnRH antagonist, relugolix, daidzein, uterine fibroids, endometriosis

## Abstract

Relugolix, a gonadotropin-releasing hormone (GnRH) receptor antagonist, has been well studied in the treatment of endometriosis symptomatic. It is mainly metabolized by the CYP3A subfamily of P450 enzymes, while minorly metabolized by CYP2C8. Daidzein in different dose groups exhibited a certain induction on the mRNA expression level of CYP3A4 and resulted in the potent induction of CYP3A4. However, it is still unknown whether daidzein and relugolix interact. We developed an effective ultra-performance liquid chromatography tandem mass spectrometry (UPLC-MS/MS) method to study the effect of daidzein on the pharmacokinetics of relugolix in rats after oral administration of 12 mg/kg relugolix in a single or mixed of 50 mg/kg daidzein. The results showed that the method had respectable linearity (*r* ^2^ > 0.999) on the scale of 0.7–1000 ng/mL. The intra-day precision was between 3.0% and 8.4% in this assay, and the inter-day was between 4.0% and 11.7%. The intra-day accuracy was from -4.3% to 6.1%, and the inter-day was 2.9% to 12.1%. Another three key indicators, including the stability, the recovery rate of extraction and the new technique’s matrix effect, were perfectly in accord with the test verification rule in the biological medium by the United States Food and Drug Administration. Meanwhile, treatment with daidzein led to a decrease in C_max_ and AUC_0–t_ of relugolix by about 15.56% and 21.36%, respectively. Although there was no statistical difference in pharmacokinetic parameters, it reflected the induction trend of daidzein on relugolix metabolism for food-drug interaction. It would provide reference and improvement value for subsequent experiments.

## Introduction

Female reproductive health is a growing area of interest. Uterine fibroids (UF) and endometriosis are estrogen-related chronic gynecological disorders and affect millions of women worldwide. UF is a common benign uterine neoplasm in females, and the prevalence increases throughout the premenopausal years, estimated at 33–77% during their reproductive years and 70–80% by menopause (Parker, 2007; Stewart et al., 2016; Baird and Harmon, 2021). Primary symptoms are pelvic pressure or pain, menorrhagia and urinary symptoms, which are related to its size, number and location in the uterus (Leppert et al., 2021). Endometriosis that characterized by endometrial and stromal tissue abnormally located outside the uterine cavity has an estimated prevalence of 14% to 35% per year in women (Sarria-Santamera et al., 2020). It is predominantly associated with chronic pelvic pain, severe dysmenorrhea, menorrhagia, dyspareunia, and infertility during reproductive age (As-Sanie et al., 2019; Zannoni et al., 2020). In previous reports, women with UF were more likely to have endometriosis than those without fibroids, which suggested that the two disorders may be associated and their etiology has many similarities (Uimari et al., 2011; Teresinski et al., 2019; Yadav et al., 2021). There are very few discoverable ideal treatment methods for the above two diseases. For UF, the common treatment options are hysterectomy and myomectomy by surgery (Schlaff et al., 2020). As drug interventions, the medication for UF is similar to endometriosis, including oral contraceptives hormones (estrogen and progestin-based) (Berlanda et al., 2016), levonorgestrel, nonsteroidal anti-inflammatory drugs (Gezer and Oral, 2015; Lewis et al., 2018), and Gonadotropin-releasing hormone (GnRH) agonists, particularly, alleviating the symptom of heavy menstrual bleeding (Dunselman et al., 2014). Nevertheless, combined oral contraceptive medications were not effective in every patient and associated with an increasing risk of thromboembolic events (Rafique and Decherney, 2017). Progestin-only products, similar to GnRH agonists and danazol, can relieve pelvic pain effectively but may increase the risk of uterine bleeding (Gezer and Oral, 2015). In addition, injectable GnRH agonist peptides, such as leuprorelin, are likely to cause a transient increase in gonadotropins secretion and finally lead to a temporary worsening of symptoms (Stewart et al., 2016; Yao et al., 2017).

**FIGURE 1:**
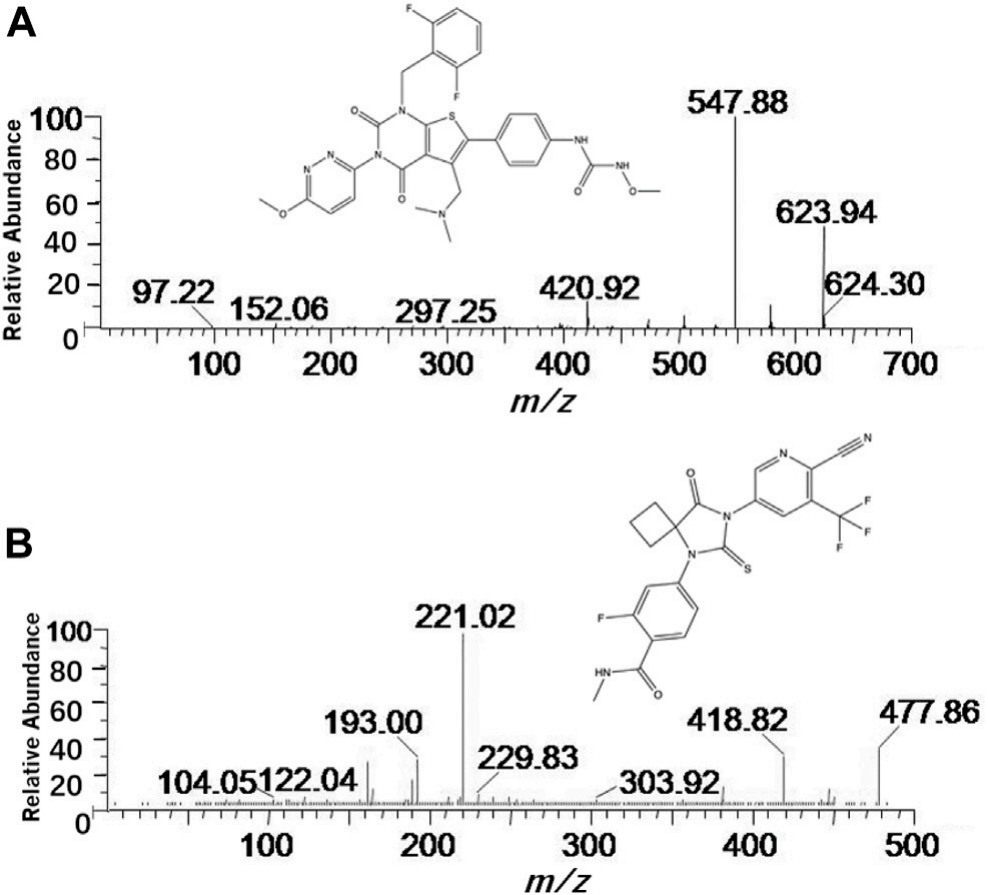
Mass spectra of relugolix **(A)** and apalutamide **(B)** in the present research.

With the potential to overcome the limits of GnRH-agonists, GnRH antagonists are characterized by causing a sustained drop of serum estrogen with dose-dependent, permitting to avoid hormonal add-back therapy and the absence of the flare effect (Chaichian et al., 2017; Pohl et al., 2020). Relugolix (Figure 1A), a newly-found non-peptide GnRH antagonist to treat the two diseases above, takes effect more rapidly than GnRH agonists developed by Takeda and ASKA Pharmaceutical (Markham, 2019). The drug suppresses the release of gonadotropin from the pituitary resulting in reduced levels of estradiol, progesterone and testosterone, without an initial rise in hormone levels (Ali et al., 2021; Relugolix, 2021). Relugolix recently has got the permission for marketing in Japan to treat symptoms associated with UF. The results of relevant studies, attempting to assess the efficacy of the drug in the treatment of endometriosis-associated pain and prostate cancer, are shown an ideal way (Dearnaley et al., 2020; Jena, 2020; Osuga et al., 2021). Relugolix may become a new oral therapy option for its significant improvements in symptoms and good tolerability, especially, in women who refused other hormonal therapies simply or contraindication.

Isoflavonoids, the subfamily of Leguminosae, a group of polyphenolic plant compounds, are particularly prevalent in the plant kingdom. Daidzein and genistein are the most ingredients in the isoflavonoids (Havsteen, 1983). Daidzein isolated from natural products, such as soybean, pasture grasses and cereals, showed biologically actives, like unique estrogenic activity and strong antioxidant activity (Cherdshewasart and Sutjit, 2008). As a dietary antioxidant, it may protect against oxidative stress, an important mechanism, linked to inflammation and macromolecule damage by free radicals and oxygen-related and N-oxidizing agents. The property of antioxidant may protect the body from hormone-related cancers, like breast, prostatic and endometrial cancerization (Miadokova, 2009; Toktay et al., 2020).

According to the literature, daidzein in different dose groups exhibited certain induction on the mRNA expression level of CYP3A4 and resulted in the potent induction of CYP3A4 (Kopecna-Zapletalova et al., 2017). Relugolix is mainly metabolized by the CYP3A subfamily of P450 enzymes, while with a minor metabolism by CYP2C8. In addition, co-administration with rifampin (P-gp and strong CYP3A inducer) decreased the AUC and C_max_ of relugolix by 55% and 23%, respectively (https://www.accessdata.fda.gov/drugsatfda_docs/label/2020/214621s000lbl.pdf).

Thus, it is important to set up a rapid and effective method for detecting relugolix concentration *in vivo*, for there is few effective monitoring method currently. In this research, we developed and validated an efficient UPLC-MS/MS method for the detection of relugolix in rat plasma. For implementation, we got the plasma samples of rats that have taken relugolix at a single dose of 12 mg/kg orally (control group) and a recombination dose of 50 mg/kg daidzein orally (experiment group) for the pharmacokinetics study. Moreover, the pharmacokinetic profiles of relugolix determined by UPLC-MS/MS would be a preponderance for further drug concentration monitoring studies including food-drug or drug-drug interactions.

## Materials and Methods

### Chemicals

The analyte relugolix required high quality (purity >98%) was provided by Beijing Sunflower and Technology Development CO, Ltd (Beijing, China), as well apalutamide selected to be the internal standard (IS). Daidzein was from Shanghai Chuangsai Technology Co., Ltd (Shanghai, China). The chemical reagent methanol and acetonitrile, as HPLC grade, purchased from Merck Company (Darmstadt, Germany). Deionized water produced by Milli-Q water purification system (Millipore, Bedford, United States) met academic reagent criterion.

### UPLC-MS/MS Conditions

The analytical work was performed on the liquid chromatograph with a 1.7 μm Acquity BEH C18 column of 2.1 mm × 50 mm, set at 40°C, as a core component of UPLC Acquity system (Waters Corp, Milford, MA, United States). The gradient elution was composed with organic phase solution of acetonitrile (A), and water phase contained 0.1% formic acid (B). The program for gradient elution (B): 90% at 0–0.5 min, 90%–10% at 0.5–1.0 min, maintain at 10% for 0.4 min, fast conversion from 10% to 90% at 1.4–1.5 min, then balance with 90% for 0.5 min. A speed of 0.30 mL per min was set for the flow while 2.0 min was carried out for a specimen circulation.

The Acquity UPLC system, equipped with an electrospray ionization (ESI) source and a Xevo TQ-S triple quadrupole mass spectrometer, was used to detect the analyte in positive ionization mode. Other than that, multiple reaction monitoring (MRM) was applied to determine the analyte with the precursor-to-product ion transitions: *m/z* 624.30 → 547.88 and *m/z* 477.86 → 221.02 for the quantification of relugolix and IS, respectively. The collision energy and cone voltage were 25 eV and 30 V for IS, 20 eV and 30 V for relugolix. The results were verified when the best conditions were set as below: the optimum desolation temperature was 600°C, the optimum capillary voltage was 1.0 kV, the optimum collision gas was 0.15 mL/min, the optimum conic gas was 150 L/h and the optimum desolation gas was 1000 L/h. Finally, the data was collected by using the Masslynx 4.1 software which was furnished on the new UPLC-MS/MS analysis system.

### Preparation of Stock Solution, Calibration Standard, and Quality Control (QC)

The exact concentration of standard substance was dissolved in methanol to obtain 1.0 mg/mL relugolix for preparing quality control (QC) and calibration curve specimens. Calibration standard for specimens was diluted from the corresponding stock solutions with methanol to gradient concentrations, as well as the dilution of IS solution to 100 ng/mL. The levels of calibration curve were 0.7–1000 ng/mL and QC samples were 1.4, 80, 800 ng/mL by adding 10 μL of the corresponding solution to 90 μL blank plasma of rat. The stock solution and working fluids were stored in a refrigerator at 4°C, and put at room temperature for at least 10 min to reduce temperature interference before the test.

### Specimen Preparatory Procedure

In this study, protein isolation method was used to prepare specimens. In short, adding 20 μL apalutamide (IS) solution into 100 μL plasma in a 2.0 mL Eppendorf tube before vortexing for 30 s, then 300 μL acetonitrile was added to precipitate the plasma protein and vortexed for 2.0 min. Samples were centrifugated 10 min at 13,000 × g to get the supernatant for the new employed UPLC-MS/MS system for the purpose of making a specific measurement.

## Method Verification

The bioanalytical validation method in this test was based on the principles and the guidelines of the China Food and Drug Administration (FDA), as well as the United States FDA, and a guiding force of the European Drug Administration also played an important role in this study (Feng et al., 2021; Zhao et al., 2021).

For the purpose of assessing the specificity of this newly developed method, plasma samples from six rats in the different batches were collected. To verify the influence from different source samples, tests of the representative chromatograms were performed during which relugolix (as analyte) and apalutamine (as IS) were set to make the certain curve with coverage area. On the calibration curved line, y represents the ratio of the peak area of relugolix to the IS, x describes the theoretical concentrations, and 1/*x* ^2^ was employed to be an important indicator as the weight factor. Moreover, the concentration of relugolix at 0.7 ng/mL was the lower limit of quantitation (LLOQ) and defined as the minimum solution concentration in the calibration curved line. It requires that relative standard deviation (RSD, %), the visual indicator for precision, need to be less than 20% and relative error (RE, %), the indicator for accuracy, is supposed to be the same requirement.

A comparison was made between the area of blank samples after extraction and methanol added at respective concentration levels to investigate the influence of matrix effect. To get the data of the recovery rate, it is an important step to calculate the ratio of the concentration of those samples before and after separation. Six comparative and repeated trials were carried out to assess the indicators above, during which the samples were set at three different QC levels (1.4, 80 and 800 ng/mL) for the purpose of ascertaining both the data of recovery rate and matrix effect. Besides, intra-day and inter-day data of both the relative standard deviation (RSD%) and relative error (RE%) were calculated with six repetitions per day for three consecutive days.

In this study, we evaluated the stability of six duplicate specimen of rat plasma with relugolix levels of 1.4, 80 and 800 ng/mL, under four probable circumstances: first test was to freeze/thaw the experimental samples completely three times; store the samples at 10°C for 3 h, room temperature for 2 h and -40°C for 28 days as the storage tests. With the work of experiments above, the assessment of stability of relugolix in rat plasma and quality of specimens was rigorous.

### Pharmacokinetic Application

In this study, the female Sprague-Dawley (SD) rats were 2 months of age (weight 200 ± 20 g) and provided from Wenzhou Medical University (Zhejiang, China). The animal experiments were adhered to its corresponding Care and Use of Laboratory Animal Regulation and Rule regards, and approved by the Animal Protection and Use Committee of Wenzhou Medical University. Water was not restricted before the experiments during fasting 12 h. As oral administration drugs of rats, daidzein and relugolix were dissolved in a 0.5% CMC-Na aqueous solution to 10 mg/mL and 2.4 mg/mL, respectively. A dose of 50 mg/kg daidzein was orally administrated for the experimental group, meanwhile the same volume of solvent was given to the control group. After 30 min, relugolix, a single dose of 12 mg/kg, was orally administrated to the experimental group and control group simultaneously. Then blood samples were collected at 0, 0.33, 0.67, 1, 1.5, 2, 3, 4, 6, 8, 12, 24, and 48 h independently, and about 300 μL were put into EP tubes with heparin anticoagulant. All the samples were separated under centrifugation at 4000 × g for 8 min immediately and kept at -40°C for later study. The pharmacokinetic parameters of relugolix *in vivo* were evaluated by Drug and Statistics (DAS) version 3.0 bought from Shanghai University of Traditional Chinese Medicine, in a non-compartmental model. Pharmacokinetic parameters between two groups were compared through independent-samples *t* test by Statistical Package for the Social Sciences (version 23.0; SPSS Inc, Chicago, IL, United States). *p* < 0.05 is statistically significant.

## Results and Discussion

### Approach Validation and Improvement

The present study corroborated a new efficient method to determinate the concentration of relugolix, which was separated from the biological plasma in rats by an effective UPLC-MS/MS study. Three improvements should be considered, including the majorization of chromatography conditions, the decrease in performing period, and the enhancement of the detectability. In order to reach a high level of precision and the minimum amount of experimental error, we used apalutamide as IS (the perfect match). The reason that acetonitrile was chosen as the organic media was due to its lower background noise level than methanol. Eventually, good separation and peak shape were obtained with 0.1% formic acid water channel and acetonitrile solution organic channel as mobile phase. In order to remove protein and potential interferences, an efficient extract preparation played a key role in preference to UPLC-MS/MS assay. Compared with the current extraction technology, researchers used an easy but resultful way rather than complicated solid-phase extraction and liquid-liquid extraction technology, which was demonstrated by predecessors (Chen et al., 2015; Liu et al., 2016; Tomai et al., 2021). A series of solvents were tested to find the most proper one for protein precipitation. Finally, because of acetonitrile’s better recovery, it was chosen.

### Selectivity and Matrix Effect

**FIGURE 2:**
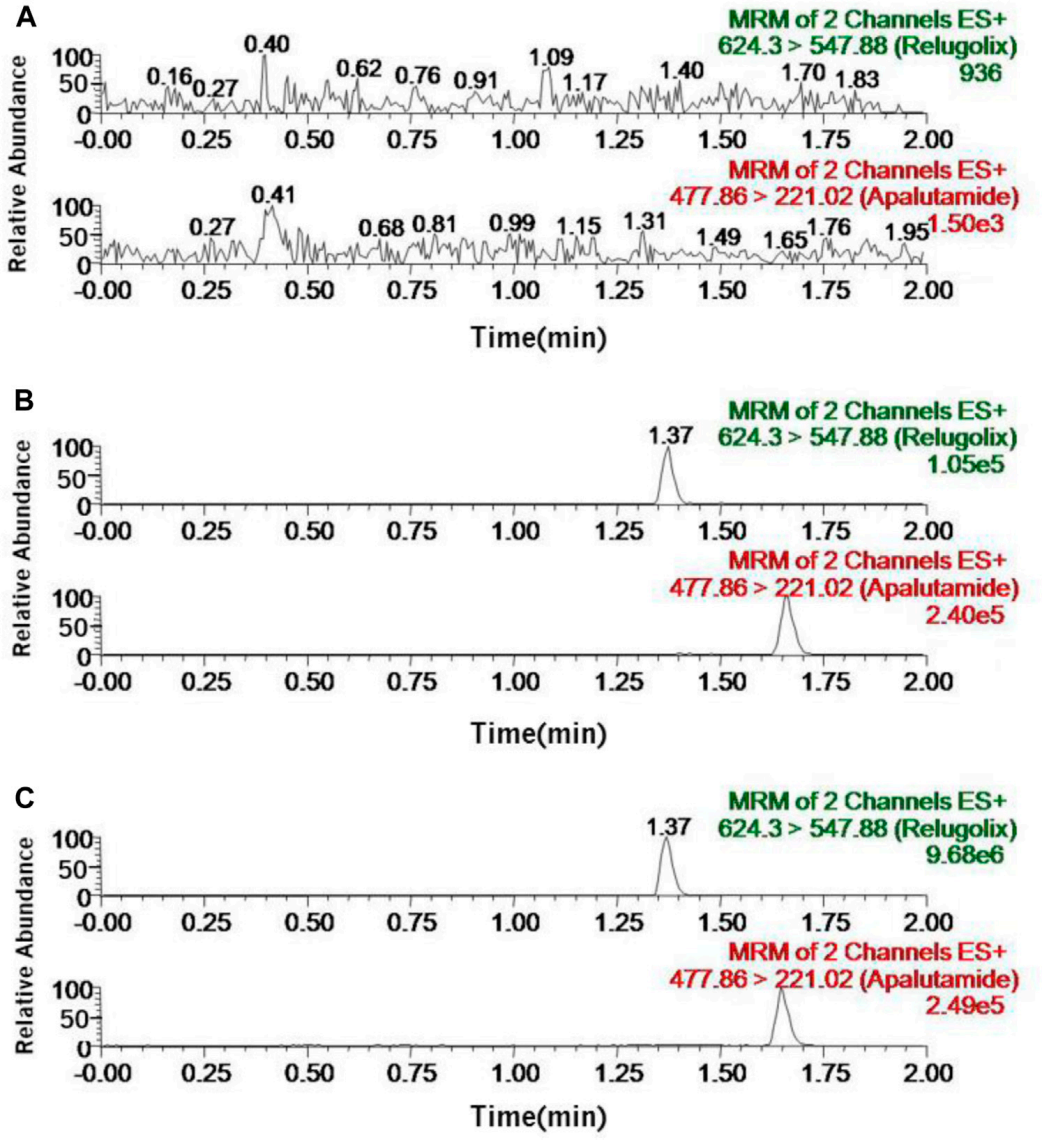
Representative chromatograms of relugolix and IS in rat plasma samples: **(A)** the blank plasma sample; **(B)** the blank plasma sample with relugolix at the LLOQ concentration (0.7 ng/mL) and IS; **(C)** the rat plasma sample after oral administration of 12 mg/kg relugolix.

Figure 2 demonstrated the selectivity of this approach. It showed the typical chromatograms of three different conditions: the sample of blank plasma (A); the blank plasma sample with relugolix at the LLOQ concentration and IS (B); the rat plasma sample of oral administration of 12 mg/kg relugolix (C). The chromatograms showed the fact that the retention time of IS was 1.65 min while the retention time of relugolix was 1.37 min, and interfering peaks induced by endogenic compounds were insignificant, which even could be neglected. At three concentration gradients of 1.4, 80 and 800 ng/mL, the matrix effect value was (112.6 ± 10.2)%, (110.7 ± 8.3)% and (108.8 ± 12.8)%, respectively, being well accepted, and interference factor of the matrix could be ignored.

### Linearity and Sensitivity

The calibration standard, which y represented as the ratio of relugolix to the IS and x as the plasma level of relugolix, was shown as follows: y = 20.3899x + 3.05209, with correlation coefficient *r* ^2^ = 0.999,935 in the scope of 0.7–1000 ng/mL. This standard of relugolix for quantification was satisfying. The LLOQ (0.7 ng/mL) was kept high exactitude and the signal to noise ratio of LLOQ was >10.

### Accuracy, Precision, and Extraction Recovery

Under 1.4, 80, 800 ng/mL concentrations that included low-to-high levels, several tests were performed for six times. All the results were presented in Table 1. The relugolix recovery rate in plasma sample was (80.5 ± 8.5)%, (86.2 ± 6.0)% and (88.3 ± 3.5)%, as the chart displayed, under the three QC levels, respectively. Moreover, the measurement of precision and accuracy was satisfied with the demands in three independent days.

**TABLE 1:** Accuracy, precision, matrix effect and recovery of QC samples of relugolix from plasma in rats (*n* = 6).

Concentration (ng/mL)	RSD (%)		RE (%)		Matrix Effect (%)	Recovery (%)
	Intra-day	Inter-day	Intra-day	Inter-day		
0.7	8.4	11.7	−4.3	8.7	—	—
1.4	7.1	8.9	0.0	10.0	112.6 ± 10.2	80.5 ± 8.5
80	5.0	6.6	6.1	12.1	110.7 ± 8.3	86.2 ± 6.0
800	3.0	4.0	−0.3	2.9	108.8 ± 12.8	88.3 ± 3.5

RSD, relative standard deviation; RE, relative error.

### Stability

The stability test was usually studied under four variable environments in the experimental process, including short period setting at ambient temperature for more than 2 h, 10°C for 3 h after sample extracted in a self-sampler, long period setting at -40°C for 28 days, and three complete freeze-thaw cycles as well. It was shown perfect stability under these conditions. All the outcomes of the study about stability of relugolix from rat plasma were exhibited in Table 2.

**TABLE 2:** Stability findings of relugolix from plasma in rats under different conditions (n = 6).

Concentration (ng/mL)	Room Temperature, 2 h		10°C, 3 h		Three Freeze-Thaw		−40°C, 28 days	
	RSD (%)	RE (%)	RSD (%)	RE (%)	RSD (%)	RE (%)	RSD (%)	RE (%)
1.4	7.8	0.7	11.7	−9.4	11.3	6.1	7.2	14.2
80	4.1	−1.0	9.9	−4.0	5.8	6.8	5.0	10.0
800	2.6	−14.2	5.8	−10.6	3.2	−3.7	3.4	−1.9

RSD, relative standard deviation; RE, relative error.

### Application of This Approach in the Pharmacokinetics

**FIGURE 3:**
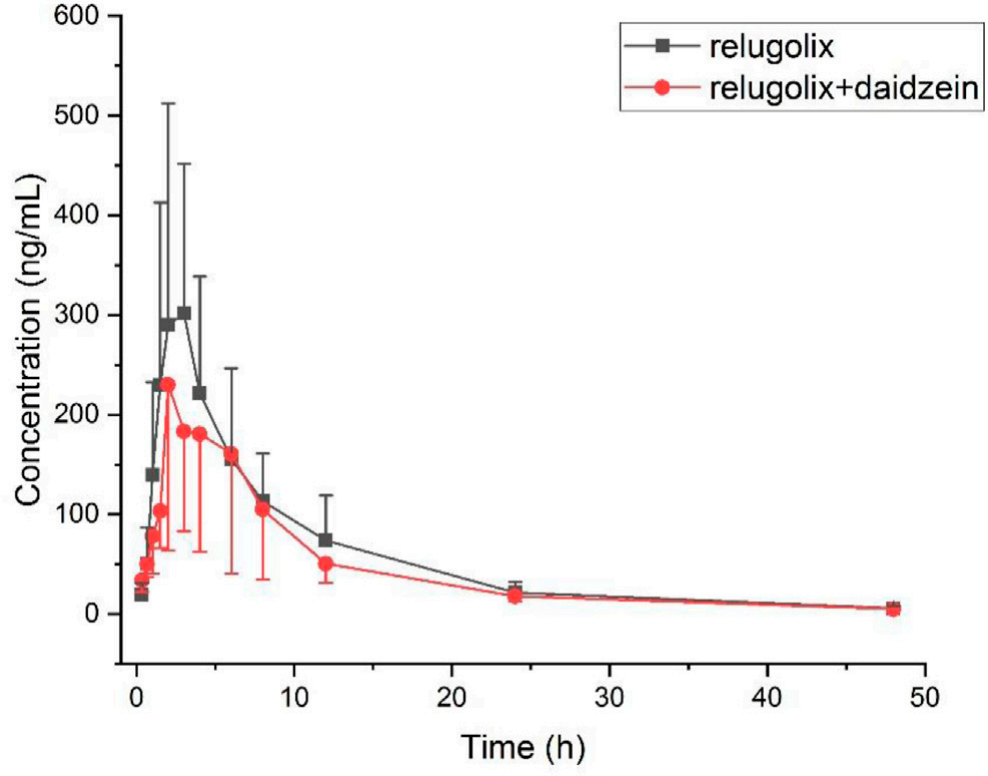
Plasma concentration versus time after oral administration of 12 mg/kg relugolix with or without daidzein in six rats for each group (Mean ± SD).

The applicability of this method was verified in a preliminary study of plasma relugolix concentration levels in rats after oral administration alone or in combination with 50 mg/kg daidzein. Conclusions of the most important pharmacokinetic parameters discovered through the test were shown in Table 3. After oral administration with a single dose of 12 mg/kg relugolix in both groups, the data of plasma concentrations that changed with time was portrayed in Figure 3.

**TABLE 3:** Main pharmacokinetic parameters after taking a single oral dose of relugolix with or without daidzein to rats (*n* = 6).

Parameters	Relugolix	Relugolix + daidzein
T_1/2_ (h)	8.95 ± 0.75	8.45 ± 1.30
T_max_ (h)	2.33 ± 0.82	2.42 ± 0.92
C_max_ (ng/mL)	350.17 ± 190.35	295.70 ± 135.01
CL (L/h•kg)	5.50 ± 3.29	6.62 ± 3.44
AUC_0→t_ (ng/mL•h)	2743.18 ± 1353.80	2157.35 ± 964.50
AUC_0→∞_ (ng/mL•h)	2813.49 ± 1390.68	2196.56 ± 975.10

T_1/2_, half-life time; C_max_, maximum plasma concentration; T_max_, time to C_max_; CL, clearance; AUC, Area under the concentration-time curve; MRT, mean residence time.

It turned out that after orally taking a certain capacity of relugolix, there was no significant statistical difference in the critical pharmacokinetic parameters. Nevertheless, it showed a tendency of induction for the experimental group in terms of AUC_0→t_ compared (2743.18 ± 1353.80) g/mL•h to (2157.35 ± 964.50) ng/mL•h; the terminal half-value elimination period (t_1/2_) compared (8.95 ± 0.75) h to (8.45 ± 1.30) h and the total clearance rate data compared (5.50 ± 3.29) L/h•kg to (6.62 ± 3.44) L/h•kg. The reason for the result without significant difference may be that: mainly, the sample sizes were too small, with only six rats in each of the experimental group and the control group, and it caused the objective deviation. From this point, we will expand the sample sizes in subsequent studies. In addition, daidzein should be stratified into different concentration gradients, and each gradient is in line with a reasonable sample size for further study. Moreover, there are certain differences between rat metabolic enzymes and human liver drug enzymes. Finally, we should design experiments to prepare human hepatocytes *in vitro* to further explore the food-drug interaction for better verification. The *in vivo* experiment in rats is of a certain reference value, which provides important pharmacokinetic parameters for reference to subsequent experiments and it also hints at the significance of subsequent experiments.

## Conclusion

Relugolix, as an effective GnRH receptor antagonistic drug, has demonstrated the optimized drug therapy effect and security when it was made use of in the process of treating bleeding in menstruation and estrogen-associated pain in UF and endometriosis. To conclude, this study is the first attempt to validate and verify a quantitative method of relugolix in rat plasma with ideal linearity in the range of 0.7–1000 ng/mL. A single dose of 12 mg/kg relugolix was administered orally to the experimental group (a combination dose of 50 mg/kg daidzein) and the control group for pharmacokinetics study, in which the new method was verified successfully as presumed. Moreover, after protein precipitation with acetonitrile, the relugolix in rat plasma could be recovered efficiently by UPLC-MS/MS method without matrix effect. This method had the characteristics of high precision, high sensitivity and high efficiency, and the detection error meets the standard range. Although there was no statistical difference in pharmacokinetic parameters, it still reflected the induction trend of daidzein on relugolix for food-drug interaction and it provided reference and improvement value for subsequent experiments.

## Data Availability

The original contributions presented in the study are included in the article/Supplementary Material, further inquiries can be directed to the corresponding authors.
